# Supplementary material for: Effect of Plant Defenses and Plant Nutrients on the Performance of Specialist and Generalist Herbivores of Datura: A Macroevolutionary Study
Source: Plants (Basel). 2023 Jul 11;12(14):2611. doi: 10.3390/plants12142611 (PMC10384791; doi:10.3390/plants12142611)
Supplement: Supplementary file 1 [file plants-12-02611-s001.zip › plants-2440397-supplementary.docx]

**SUPPLEMENTARY MATERIAL**

**Effect of plant defenses and plant nutrients on performance of specialist and generalist herbivores of Datura: a Macroevolutionary study**

| **TABLE S1**. GenBank accession number of sequences obtained for species of *Datura* (see text). | | | | |
| --- | --- | --- | --- | --- |
| **Genus** | **Species** | **Accession number** | | |
|  |  | ***psb*A-*trn*H** | ***trn*L-*trn*F** | **rRNA ITS** |
| *Datura* | *wrightii* | JX467618 | JX467585 | JX467602 |
| *Datura* | *lanosa* | JX467619 | JX467586 | JX467603 |
| *Datura* | *metel* | JX467620 | JX467587 | JX467604 |
| *Datura* | *discolor* | JX467621 | JX467588 | JX467605 |
| *Datura* | *reburra* | JX467622 | JX467589 | JX467606 |
| *Datura* | *inoxia* | JX467623 | JX467590 | JX467607 |
| *Datura* | *kymatocarpa* | JX467625 | JX467592 | JX467609 |
| *Datura* | *pruinosa* | JX467626 | JX467593 | JX467610 |
| *Datura* | *stramonium* | JX467627 | JX467594 | JX467611 |
| *Datura* | *quercifolia* | JX467628 | JX467595 | JX467612 |
| *Datura* | *ferox* | JX467629 | JX467596 | JX467613 |

| **TABLE S2**. Variation in food consumption (FC); cm^2^, biomass increment (BI); g, damaged leaves (DL); number, and growth efficiency (GE); g/cm^2^ between the specialist herbivore (S) *Lema trilineata* *daturaphila* and the generalist herbivore (G) *Spodoptera frugiperda* in each *Datura* spp. | | | | | | | | | | |
| --- | --- | --- | --- | --- | --- | --- | --- | --- | --- | --- |
|  | **FC** | | | | **BI** | | | | | |
| **Sp** | $\bar{x}$_(S )_$\pm$ se | $\bar{x}$_(G)_$\pm$ se | ***F*** | ***P*** | $\bar{x}$_(S )_$\pm$ se | $\bar{x}$_(G)_$\pm$ se | | ***F*** | ***P*** |  |
| ***discolor*** | 1.64(0.24) | 0.41(0.24) | 3.61 | **<0.0009** | 0.48(0.098) | -0.01(0.098) | | 3.57 | **0.001** |  |
| ***ferox*** | 0.71(0.25) | 1.03(0.25) | -0.89 | 0.378 | -0.0003(0.038) | | -0.13(0.038) | 2.49 | **0.017** |  |
| ***inoxia*** | 0.63(0.16) | 0.71(0.16) | -0.34 | 0.73 | 0.16(0.14) | -0.12(0.14) | | 1.36 | 0.18 |  |
| ***kymatocarpa*** | 0.8(0.14) | 0.52(0.14) | 1.38 | 0.17 | 0.52(0.096) | -0.083(0.096) | | 4.42 | **< 0.0001** |  |
| ***lanosa*** | 2.39(0.24) | 0.95(0.24) | 4.16 | **0.0002** | 1.2(0.12) | 0.067(0.12) | | 6.71 | **< 0.0001** |  |
| ***metel*** | 1.58(0.23) | 0.67(0.23) | 2.73 | **0.009** | 0.7(0.14) | 0.16(0.14) | | 2.73 | **0.009** |  |
| ***pruinosa*** | 1.17(0.17) | 0.38(0.17) | 3.14 | **0.003** | 1.24(0.15) | -0.19(0.15) | | 6.64 | **< 0.0001** |  |
| ***quercifolia*** | 0.76(0.25) | 2.17(0.25) | -3.9 | **0.0004** | 0.4(0.13) | 0.47(0.13) | | -0.35 | 0.72 |  |
| ***reburra*** | 1.48(0.17) | 0.18(0.17) | 5.17 | **< 0.0001** | 2.04(0.25) | -022(0.25) | | 6.23 | **< 0.0001** |  |
| ***stramonium*** | 0.98(0.14) | 1.18(0.14) | -0.93 | 0.35 | 0.34(0.05) | 0.09(0.05) | | 3.07 | **0.003** |  |
| ***wrightii*** | 2.4(0.24) | 0.68(0.24) | 4.61 | **< 0.0001** | 1.26(0.17) | 0.03(0.17) | | 4.9 | **< 0.0001** |  |
| **Sp** | **DL** | | | | **GE** | | | | | |
| ***discolor*** | 2.0(0.29) | 1.95(0.29) | 0.12 | 0.9 | 0.26(0.18) | -0.03(0.18) | | 1.13 | 0.26 |  |
| ***ferox*** | 2.05(0.27) | 2.45(0.27) | -1.04 | 0.3 | -0.001(0.002) | -0.012(0.002) | | 3.66 | **0.0008** |  |
| ***inoxia*** | 1.0(0.18) | 2.4(0.18) | -5.27 | **< 0.0001** | -0.005(0.04) | -0.074(0.04) | | 1.19 | 0.24 |  |
| ***kymatocarpa*** | 1.6(0.2) | 2.25(0.2) | -2.29 | **0.027** | 0.005(0.003) | -0.018(0.003) | | 5.39 | **< 0.0001** |  |
| ***lanosa*** | 1.9(0.17) | 2.6(0.17) | -2.75 | **0.009** | 0.005(0.003) | 0.001(0.003) | | 0.74 | 0.46 |  |
| ***metel*** | 1.55(0.22) | 2.55(0.22) | -3.09 | **0.003** | 0.006(0.007) | 0.02(0.007) | | -1.29 | **0.2** |  |
| ***pruinosa*** | 3.8(0.46) | 2.7(0.46) | 1.69 | 0.09 | 0.02(0.014) | -0.059(0.014) | | 3.83 | **0.0005** |  |
| ***quercifolia*** | 2.6(0.21) | 4.15(0.21) | -5.08 | **< 0.0001** | 0.011(0.005) | 0.012(0.005) | | -0.1 | 0.92 |  |
| ***reburra*** | 4.2(0.42) | 2.3(0.42) | 3.14 | **0.003** | 0.0006(0.075) | -0.17(0.075) | | 1.74 | 0.08 |  |
| ***stramonium*** | 2.95(0.28) | 2.7(0.28) | 0.62 | 0.53 | 0.006(0.005) | -0.001(0.005) | | 1.11 | 0.27 |  |
| ***wrightii*** | 1.7(0.19) | 1.9(0.19) | -0.74 | 0.46 | 0.003(0.023) | -0.02(0.023) | | 0.97 | 0.33 |  |
| Significant values (*P*< 0.05) are indicated in bold type. | | | | | | | | | | |

| **TABLE S3.** Akaike´s information criterion (AIC) values for 88 statistical models of plant nutrient (N; nitrogen, P; phosphorous, C; carbon, and W; water) and defenses (hyos; hyoscyamine, scop; scopolamine, totalk; total alkaloids, and trico; leaf trichomes)*.* In bold is indicated the best model (the one with the lowest AIC) for each herbivore performance variable. | | |
| --- | --- | --- |
| **Statistical model for plant nutrients** | **AIC value of “S”** | **AIC value of “G”** |
| Biomass increment (BI) | | |
| N+P+C+W | -7.51 | -28.15 |
| N+C+W | -6.92 | -29.97 |
| N+P+W | -7.07 | -24.41 |
| N+C+P | -9.503 | -20.36 |
| P+C+W | -9.51 | -29.71 |
| N+P | -9.066 | -19.81 |
| N+C | -8.81 | -22.32 |
| N+W | -7.142 | -26.21 |
| P+C | -9.014 | -25.66 |
| P+W | -8.903 | -21.44 |
| C+W | **-11.496** | **-31.43** |
| Food consumption (FC) | | |
| N+P+C+W | -16.21 | -17.44 |
| N+C+W | -11.8 | -16.1 |
| N+P+W | -17.32 | -16.81 |
| N+C+P | -17.91 | -13.02 |
| P+C+W | -18.09 | -19.41 |
| N+P | -19.02 | -13.94 |
| N+C | -13.17 | -14.58 |
| N+W | -13.22 | -18.75 |
| P+C | **-19.9** | -18.71 |
| P+W | -19.29 | -12.31 |
| C+W | -13.8 | **-20.75** |
| Growth efficiency (GE) | | |
| N+P+C+W | -38.17 | -47.21 |
| N+C+W | -40.09 | **-48.8** |
| N+P+W | -37.81 | -42.2 |
| N+C+P | **-40.17** | -46.6 |
| P+C+W | -39.85 | -45.3 |
| N+P | -19.02 | -43.3 |
| N+C | -13.17 | -48.38 |
| N+W | -13.22 | -43.6 |
| P+C | -19.9 | -43.62 |
| P+W | -19.29 | -46.57 |
| C+W | -13.8 | -47.06 |
| Damaged leaves (DL) | | |
| N+P+C+W | -9.6 | -37.15 |
| N+C+W | -11.55 | -34.46 |
| N+P+W | 11.39 | -39.15 |
| N+C+P | -11.39 | -26.58 |
| P+C+W | -12.72 | -38.77 |
| N+P | -8.25 | -28.58 |
| N+C | **-13.37** | -25.43 |
| N+W | -12.83 | -36.46 |
| P+C | -9.98 | **-40.75** |
| P+W | -9.24 | -27.26 |
| C+W | -10.05 | -36.42 |
|  |  |  |
|  |  |  |
| **Table S3, continuation.**  **Statistical model for plant defenses** | **AIC value of “S”** | **AIC value of “G”** |
| Biomass increment (BI) | | |
| hyos+scop+totalk+trico | -7.29 | -18.3 |
| hyos+totalk+trico | -6.31 | -19.27 |
| hyos+scop+totalk | -6.87 | -18.89 |
| hyos+scop+trico | -7.95 | -18.46 |
| scop+totalk+trico | -9.23 | -19.85 |
| hyos+scop | -8.34 | -19.79 |
| hyos+totalk | -7.96 | -20.06 |
| hyos+trico | -7.147 | -20.59 |
| scop+totalk | -8.67 | -20.46 |
| scop+trico | **-9.87** | -20.84 |
| totalk+trico | -7.29 | **-21.22** |
| Food consumption (FC) | | |
| hyos+scop+totalk+trico | -11.35 | -11.21 |
| hyos+totalk+trico | -13.34 | -10.91 |
| hyos+scop+totalk | -12.22 | -10.69 |
| hyos+scop+trico | -13.35 | -10.83 |
| scop+totalk+trico | -12.69 | **-13.19** |
| hyos+scop | -14.17 | -12.63 |
| hyos+totalk | -14.19 | -12.81 |
| hyos+trico | **-15.27** | -10.2 |
| scop+totalk | -13.38 | -10.9 |
| scop+trico | -14.56 | -11.83 |
| totalk+trico | -14.43 | -12.1 |
| Growth efficiency (GE) | | |
| hyos+scop+totalk+trico | -39.25 | -46.6 |
| hyos+totalk+trico | -39.74 | -44.1 |
| hyos+scop+totalk | -39.82 | -48.04 |
| hyos+scop+trico | -39.88 | -41.74 |
| scop+totalk+trico | -38.64 | -47.52 |
| hyos+scop | -40.48 | -43.21 |
| hyos+totalk | -40.47 | -43.35 |
| hyos+trico | **-41.68** | -46.105 |
| scop+totalk | -40.12 | -43.73 |
| scop+trico | -40.06 | **-48.96** |
| totalk+trico | -40.02 | -48.11 |
| Damaged leaves (DL) | | |
| hyos+scop+totalk+trico | -17.79 | -27.54 |
| hyos+totalk+trico | -16.53 | -29.52 |
| hyos+scop+totalk | -15.99 | -28.62 |
| hyos+scop+trico | -18.49 | -29.54 |
| scop+totalk+trico | -17.2 | -29.32 |
| hyos+scop | -17.92 | -30.58 |
| hyos+totalk | -16.9 | -31.52 |
| hyos+trico | -11.82 | -23.96 |
| scop+totalk | -17.06 | -31.27 |
| scop+trico | **-20.33** | **-30.61** |
| Correlations between components of herbivore performance (food consumption; FC, biomass increment; BI, DL; leaf damaged, and GE; growth efficiency) of specialist herbivore *Lema trilineata daturaphila* (“S”) and generalist *Spodoptera frugiperda* (“G”), and plant traits were corrected by phylogenetic relationships (PGLs) of *Datura* | | |


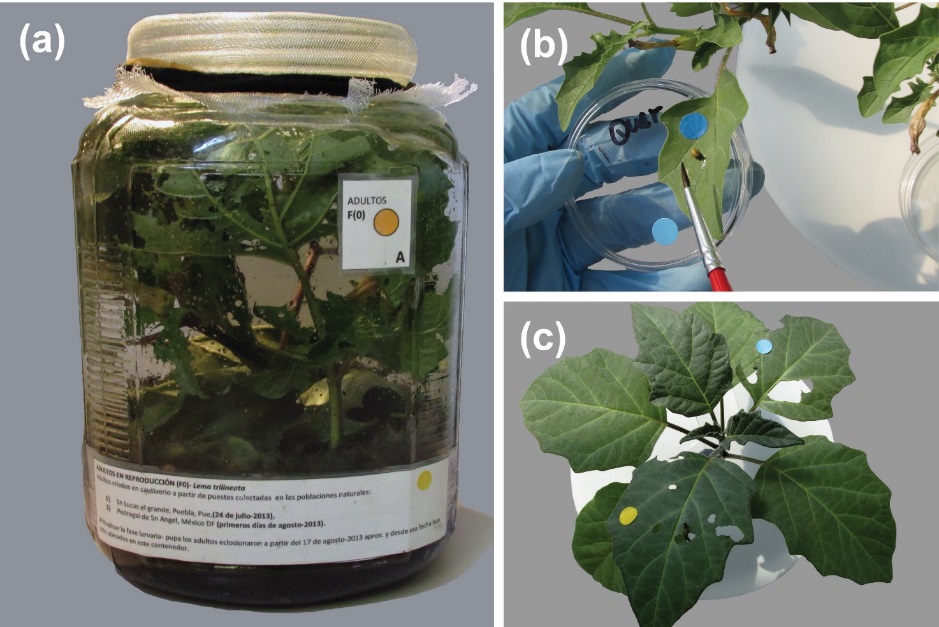


**FIGURE S1**. Experiment of the specialist herbivore *Lema trilineata daturaphila*. (a) Rearing of larvae, (b) setting of larvae at the adaxial side of the leaf, and (c) leaf marking and consumption by larvae.


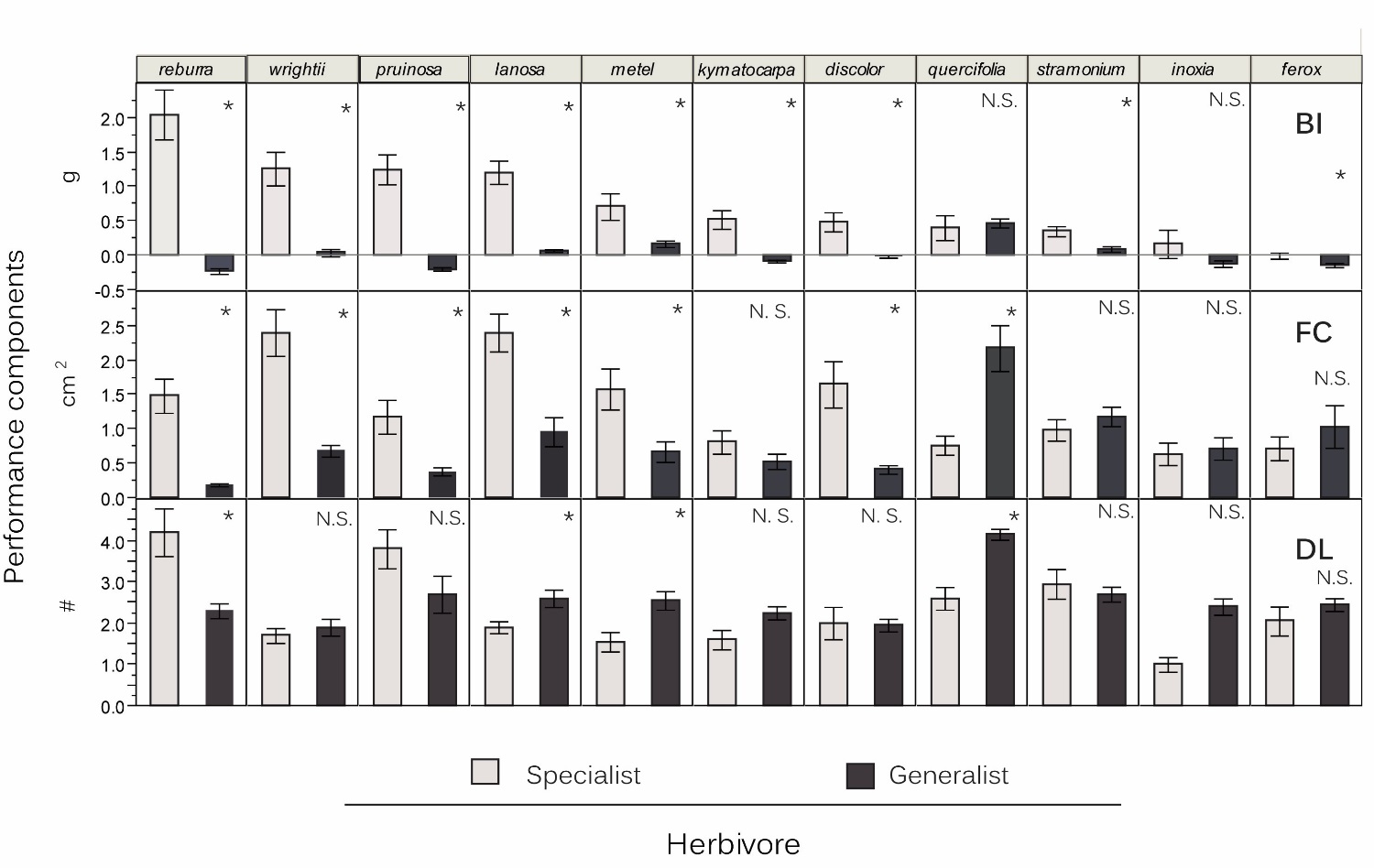


**FIGURE S2**. Variation of BI (biomass increment; g), FC (food consumption; cm^2^), and DL (damaged leaves; number), between the specialist herbivore *Lema trilineata* *daturaphila* and the generalist herbivore *Spodoptera frugiperda* across *Datura* spp. (*, *P*< 0.05).
